# Genetically Estimated Ancestry and the Risk of Pre-Eclampsia: A Multiethnic Case-Control Study

**DOI:** 10.1016/j.jacadv.2025.102283

**Published:** 2025-11-03

**Authors:** Frances Conti-Ramsden, Kypros Nicolaides, Argyro Syngelaki, Peter Dixon, Catherine Williamson, Maria Tsoukaki, Alan Wright, Lucy C. Chappell, Pirro G. Hysi, Antonio de Marvao

**Affiliations:** a Department of Women and Children’s Health, School of Life Course & Population Sciences, King’s College London, London, United Kingdom; b Harris Birthright Research Centre for Fetal Medicine, Fetal Medicine Research Institute, King’s College London, London, United Kingdom; c Department of Metabolism, Digestion and Reproduction, Institute of Reproductive and Developmental Biology, Imperial College London, London, United Kingdom; d Department of Twin Research and Genetic Epidemiology, King's College London, London, United Kingdom; e UCL Great Ormond Street Institute of Child Health, University College London, London, United Kingdom; f Sørlandet Sykehus Arendal, Arendal, Norway; g Myriad High Computing Performance System, University College London, London, United Kingdom; h Institute of Health Research, University of Exeter, Exeter, United Kingdom; i British Heart Foundation Centre of Research Excellence, School of Cardiovascular and Metabolic Medicine and Sciences, King's College, London, United Kingdom; j Medical Research Council Laboratory of Medical Sciences, Imperial College London, London, United Kingdom

**Keywords:** ethnicity, hypertension, pre-eclampsia, pregnancy

## Abstract

**Background:**

Maternal self-reported ethnicity (SRE) is associated with pre-eclampsia (PE) risk and is included in prediction models.

**Objectives:**

The objectives of the study were to examine whether genetic ancestry estimates are associated with PE and improve the FMF (Fetal Medicine Foundation) PE risk model performance.

**Methods:**

PE cases and matched controls from the Harris-Birthright Cohort were genotyped using the Illumina Global Screening Array. Genetic ancestries were estimated using a multiethnic reference panel. African genetic ancestries were incorporated into logistic regression models alongside established clinical risk factors. Area under receiver operating characteristic curves of FMF models with and without genetic ancestries was compared.

**Results:**

This case-control study included 5,207 women: 3,513 White (1,382 PE cases, 2,131 controls) and 1,694 Black SRE (745 PE cases, 949 controls). Ethnicity-ancestry discrepancy was present in 11.3% of self-reporting Black and 5.3% of self-reporting White individuals. Higher West African genetic ancestry was independently associated with increased PE odds. Among self-reporting White women, those with 50% to 100% West African (AFR) ancestry had higher risk vs those with <5% (adjusted OR: 6.46; 95% CI: 3.37 to 12.98; *P* < 0.001). In self-reporting Black women, lower West AFR ancestry (50% to 84.9%) reduced risk vs those with 85% to 100% (adjusted OR: 0.60; 95% CI: 0.45-0.80; *P* < 0.001). Incorporating genetic ancestry did not improve FMF model performance.

**Conclusions:**

SRE imperfectly represents genetic ancestry in multiethnic populations. West AFR ancestry independently associates with the PE risk, supporting biological relevance of ancestry-based stratification. However, genetic ancestry did not improve the gold-standard clinical prediction model performance. Large genomic studies in multiethnic cohorts are needed to delineate the genetic architecture of PE.

Pre-eclampsia (PE) affects 3% to 5% of pregnant women worldwide and is associated with substantial maternal and neonatal mortality and morbidity, including maternal stroke, preterm birth, low birth weight, and stillbirth.^1^ In the absence of effective treatment options for PE beyond the delivery of the placenta, early initiation of aspirin prophylaxis, ideally by 16 weeks of pregnancy,^2^ is the cornerstone of clinical management, underscoring the need for accurate risk prediction in early pregnancy.

Epidemiological studies have consistently shown that the incidence and severity of PE varies across maternal ethnic groups, with women of Black ethnic backgrounds at highest risk.^3^, ^4^, ^5^, ^6^, ^7^ Black maternal ethnic background is currently recognized as a moderate risk factor for PE according to ACOG (American College of Obstetricians and Gynaecologists) guidelines, which recommend aspirin prophylaxis for women with 1 high or 2 or more moderate risk factors.^1^ The FMF (Fetal Medicine Foundation) PE prediction algorithm,^8^, ^9^, ^10^, ^11^ which combines established maternal clinical risk factors (such as maternal age, chronic hypertension, diabetes, chronic kidney disease, and in vitro fertilization) with ultrasound and biochemical parameters,^12^^,^^13^ also currently includes self-reported maternal ethnicity (FMF algorithm categories: Black, East Asian [EAS], Mixed–ethnicities not specified, White, and South Asian [SAS]).

However, the use of maternal ethnicity in PE risk prediction is a subject of debate.^14^ Ethnic categories are sociocultural constructs with a complex relationship with ancestry,^15^^,^^16^ and may be proxies for primarily social—as opposed to biological—determinants of health. In keeping with this, we have shown that self-reported maternal ethnicity is an imperfect proxy for genetic estimates of individual ancestry in a contemporaneous, urban antenatal population in the United Kingdom.^17^ Furthermore, we demonstrated that genetic individual pan-African (AFR) ancestry estimates are associated with the risk of early-onset PE (PE with delivery <34^+0^ weeks’ gestation) independently of self-reported maternal ethnicity and established clinical risk factors.^17^

In this study, building on these findings, we further examined the association between the genetically estimated AFR ancestry and PE risk in a large, multiethnic UK cohort, hypothesizing that AFR genetic ancestry estimates are independently associated with PE risk. Specifically, we investigated the following: 1) the association between AFR genetic individual ancestry estimates and PE and 2) whether incorporating AFR genetic ancestry estimates improves the predictive performance of the FMF PE prediction algorithm, in women of self-reported White and Black ethnic backgrounds.

## Methods

All research samples and data were derived from the Harris-Birthright Cohort (HBC). The HBC is an ongoing observational cohort study recruiting pregnant women from King's College Hospital, London, and Medway Maritime Hospital, Kent, early in pregnancy (median gestational age at enrollment 12.8 [quartile 1 (Q1) – quartile 3 (Q3): 12.4 to 13.2] weeks) with prospective, longitudinal assessments at 12- and 20-weeks’ gestation of pregnancy. The study received ethical approval from the London–Dulwich Research Ethics Committee (REC: 02-03-033, IRAS:89351). Following informed consent, maternal characteristics, blood pressure (BP), ultrasound measurements, and blood samples were taken at each assessment visit. Maternal ethnicity was self-reported. Women were asked to choose one of Black, EAS, Mixed, SAS, and White ethnic groups. Self-reported ethnicity (SRE) categories were cross-checked against ethnic groups recorded in electronic health records (recorded in ∼80% of women) and were highly congruent (1.0% discrepancy between self-reported HBC ethnic group and ethnic group recorded in electronic health record). Pregnancy outcomes including PE were recorded by clinicians following a detailed review of medical notes, laboratory investigations, and ultrasound data. A diagnosis of PE was based on the established criteria of new-onset hypertension or chronic hypertension and at least one of the following: proteinuria (≥300 min/24 h; protein to creatinine ratio ≥30 mg/mmol; ≥2+ proteinuria on dipstick testing), renal insufficiency with serum creatinine >1.1 mg/dL (97 μmol/L) in the absence of underlying renal disease, hepatic dysfunction with blood concentration of transaminases more than twice the upper limit of normal (≥65 IU/L for HBC laboratory), thrombocytopenia (platelet count <100 000/μL), neurological complications (eg, cerebral or visual symptoms), or pulmonary edema.^18^ In women who had consented to genetic studies, PE cases were matched to one or more controls without chronic hypertension who had not developed PE by the time of delivery, with matching by self-reported maternal ethnicity and date of recruitment. Women of self-reported Black and White ethnic backgrounds only, the most prevalent ethnic backgrounds in the HBC, and the most relevant to the investigation of AFR genetic ancestry were included in this study. These ethnic categories were defined according to the groupings in the source data (HBC ethnic categories), consistent with UK census ethnic groupings.^19^

### Sample processing and genotyping

Following venepuncture and centrifugation, buffy coat layers were aliquoted into 2 × 0.5 mL Eppendorf tubes and stored at −80 °C until the time of analysis. DNA was extracted, quantified, and normalized from buffy coat samples and genotyped using the Illumina Infinium Global Screening Array (version 3.0) with multidisease add-on in 2 batches. The Infinium Global Screening Array contains approximately 700,000 variants and is optimized for genome-wide coverage and performance across multiethnic populations.^20^

We used Illumina’s proprietary GenCall algorithm implemented in Genome Studio 2.0 to cluster genotypes. Sample and variant quality control filters were applied to standards recommended for Genome-Wide Association Studies.^21^ Samples were filtered for cryptic relatedness and discrepancies between reported and genetically ascertained sex. Autosomal single nucleotide polymorphisms were filtered for minor allele frequency ≥1% and <5% missingness across samples. Population-aggregated Hardy-Weinberg equilibrium (combined *P* value threshold 1 × 10^−4^) was used to select high-quality markers. Quality control steps were performed in PLINK 1.9 (www.cog-genomics.org/plink/1.9/).^22^

### Genetic individual ancestry estimates

Genetic individual ancestry estimates were calculated using a supervised model in ADMIXTURE software.^23^^,^^24^ Genotype data from the study individuals were merged with genotype data from phase 3 of the 1000 Genomes Project.^25^ As the ADMIXTURE algorithm does not take linkage disequilibrium into account,^24^ approximately 10,000 fully independent ancestry-informative marker single nucleotide polymorphisms defined in our previous study were selected for ancestry analysis.^17^ Individuals from a single 1,000 Genome Project ancestral population group, for example, Yoruban, were set as a reference group in the ADMIXTURE population model, with the putative number of ancestral populations (K) set at the total number of population labels (K = 26, Supplemental Table 1). Following this, individual reference population ancestry proportions were calculated by ADMIXTURE software for the nonreference (study group case and control) individuals.

For ease of analysis and interpretation, genetic individual ancestry estimates from the 1000 Genomes reference population (eg, Yoruban population ancestry, represented by individuals of Yoruban ethnic group from Idaban, Nigeria) were aggregated into 5 broad superpopulation groups (AFR, Admixed American [AMR], EAS, European [EUR], and SAS). AFR population groups were further split into regional AFR population groups given the high diversity of AFR populations (West AFR: Yoruban in Nigeria; Esan in Nigeria; Mende in Sierra Leone. East AFR: Luhya in Kenya) (Supplemental Table 1). For each individual, genetic ancestry estimates were expressed as percentages of total ancestry. To facilitate visualization, genetic ancestry estimates percentages were categorized into groups (0%-24.9%, 25%-49.9%, 50%-74.5%, and ≥75%).

### Fetal medicine foundation pre-eclampsia risk calculation

Prior and posterior risk of PE using the existing FMF competing risks algorithm for each individual was calculated using clinical, biochemistry, and ultrasound data recorded in the Harris-Birthright data set as previously described.^8^^,^^10^^,^^11^ The FMF competing risks algorithm calculates a prior risk based on maternal past medical history, and risk factors; maternal age, body mass index (BMI), self-reported ethnic background, parity (meaning the number of previous pregnancies), history of PE in previous pregnancies, chronic hypertension, diabetes mellitus, systemic lupus erythematosus or antiphospholipid syndrome diagnosis, and the use of assisted reproductive technologies. A posterior risk for PE was calculated at 11^+0^–14^+1^ weeks’ gestation using maternal mean arterial pressure (MAP), uterine artery pulsatility index and maternal serum pregnancy-associated plasma protein-A.

### Statistical analysis

Baseline maternal characteristics and pregnancy outcomes are presented as mean (SD) or median [Q1 – Q3] as appropriate for continuous variables and count (percentage) for categorical data. Genetic individual ancestry estimates and self-reported maternal ethnicity (ethnicity) were compared in women of Black and White self-reported backgrounds through visual inspection of ancestry proportion, Sankey plots, and cross-tabulation. Discrepancy between maternal SRE and superpopulation genetic individual ancestry estimates was primarily defined as women having <50% of the superpopulation genetic ancestry (AFR, AMR, EAS, EUR or SAS) from the group expected from their self-reported ethnic background. As there are no clear guidelines on ethnicity-genetic ancestry discrepancy in the literature to our knowledge, expected genetic ancestry thresholds of <75% and <25% are also reported.

In view of the epidemiological association between the Black ethnic group and PE, association and prediction analyses focused on AFR genetic ancestry estimates only. The association between AFR genetic individual ancestry estimates and PE was assessed separately in women of Black and White self-reported backgrounds using logistic regression models to allow for different effects across ethnic groups. Given the high genetic diversity in AFR and AFR heritage populations, regional (West and East AFR) ancestries were included in models. AFR genetic individual ancestry estimates were treated as ordinal categorical variables into models for ease of interpretation, with inspection of the ancestry distribution in self-reported Black and White individuals used to select appropriate reference categories. To assess whether there was any association between AFR genetic ancestry estimates and PE independently of established risk factors, genetic ancestry estimates were evaluated in unadjusted models, and then using models adjusted for MAP, maternal age, BMI, smoking status, conception method (in vitro fertilization and ovulation induction), diabetes, previous PE, and family history of PE. All logistic regression model results are presented as ORs or adjusted ORs (aORs) with 95% CIs. Logistic regression assumptions were assessed by examining residual plots, testing linearity of the logit using scatter plots of logit vs continuous predictors, and evaluating multicollinearity through variance inflation factors. Sensitivity analyses were conducted excluding women with highly discrepant ethnicity ancestry (self-reported White women with <10% EUR genetic ancestry; self-reported Black women with <10% AFR genetic ancestry).

We then sought to determine whether prediction of PE could be improved through addition of AFR genetic individual ancestry estimates to the established FMF risk model. To establish the baseline performance of the FMF competing risk algorithm in the cohort, FMF model performance, using FMF calculated priors or posteriors directly as predictions, was assessed by area under receiver operator characteristic curve (AUROC or C-statistic) stratified by SRE. The impact of adding AFR genetic individual ancestry estimates (as continuous variables) to model AUROC was assessed with formal statistical testing using a 2-sided Delong test. Exploratory analysis was further performed in individuals with discrepancy between SRE and majority genetic ancestry (ie, self-reported White ethnicity and <50% EUR genetic ancestry or SRE Black and <50% AFR genetic ancestry). Complete case analysis was performed for all models. A *P* value <0.05 was considered statistically significant. All data cleaning and analyses was carried out in R (version 4.4.2).^26^

## Results

Of 5,675 individuals genotyped, 5,210 individuals remained after genomic quality control steps and 5,207 individuals had complete covariate and outcome data (2,127 cases and 3,080 controls) (Supplemental Figure 1). Baseline characteristics and pregnancy outcomes stratified by SRE and case-control status are shown in Table 1. In women of both Black and White SRE, cases of PE had higher BMI and higher percentage of pre-existing diabetes and chronic hypertension than controls. Cases of PE also had lower gestational age at delivery and higher percentage of stillbirths across maternal ethnic groups. All results and analyses are reported with stratification by the self-reported maternal ethnic group (Central Illustration).

**Table 1:** Baseline Characteristics and Pregnancy Outcomes in Pre-Eclampsia Cases and Controls

	White (n = 3,513)		Black (n = 1,694)		*P* Value
	Pre-Eclampsia (n = 1,382)	Controls (n = 2,131)	Pre-Eclampsia (n = 745)	Controls (n = 949)	
Baseline characteristics					
Age, y	31.81 ± 5.8	33.18 ± 4.6	31.44 ± 6.2	30.98 ± 5.5	<0.001
Body mass index (BMI), kg/m^2^	27.79 ± 6.4	24.54 ± 4.4	30.50 ± 6.7	27.68 ± 5.6	<0.001
Smoker	78 (5.6)	48 (2.3)	14 (1.9)	22 (2.3)	<0.001
Diabetes mellitus					<0.001
Type 1 Diabetes mellitus	21 (1.5)	4 (0.2)	4 (0.5)	2 (0.2)	
Type 2 Diabetes mellitus	12 (0.9)	17 (0.8)	23 (3.1)	26 (2.7)	
Chronic hypertension	91 (6.6)	0 (0.0)	141 (18.9)	6 (0.6)	<0.001
Conception					<0.001
Spontaneous	1,262 (91.3)	1,962 (92.1)	721 (96.8)	936 (98.6)	
In vitro fertilization	101 (7.3)	150 (7.0)	14 (1.9)	12 (1.3)	
Ovulation drugs	19 (1.4)	19 (0.9)	10 (1.3)	1 (0.1)	
Previous pre-eclampsia					<0.001
Nulliparous	970 (70.2)	1,212 (56.9)	341 (45.8)	370 (39.0)	
Multiparous: no pre-eclampsia	265 (19.2)	887 (41.6)	288 (38.7)	556 (58.6)	
Multiparous: previous pre-eclampsia	147 (10.6)	32 (1.5)	116 (15.6)	23 (2.4)	
Family history of pre-eclampsia	147 (10.6)	80 (3.8)	72 (9.7)	54 (5.7)	<0.001
Mean arterial pressure (early pregnancy), mm Hg	93.42 ± 8.5	86.01 ± 7.1	95.60 ± 10.0	87.09 ± 6.7	<0.001
Pregnancy outcomes					
Gestational age at delivery, wks	38.70 (37.3-40.1)	40.00 (39.1-40.9)	38.10 (36.3-39.4)	39.90 (39.0-40.6)	<0.001
Neonatal outcome					<0.001
Neonatal death	2 (0.1)	2 (0.1)	0 (0.0)	0 (0.0)	
Stillbirth	13 (0.9)	3 (0.1)	20 (2.7)	1 (0.1)	
Early-onset pre-eclampsia (delivery <34 wks’ gestation)	124 (9.0)	0 (0.0)	115 (15.4)	0 (0.0)	<0.001
Preterm pre-eclampsia (delivery <37 wks’ gestation)	285 (20.6)	0 (0.0)	232 (31.1)	0 (0.0)	<0.001

Values are mean ± SD, n (%), or median (25th-75th percentiles), unless otherwise indicated.

**Central Illustration:**
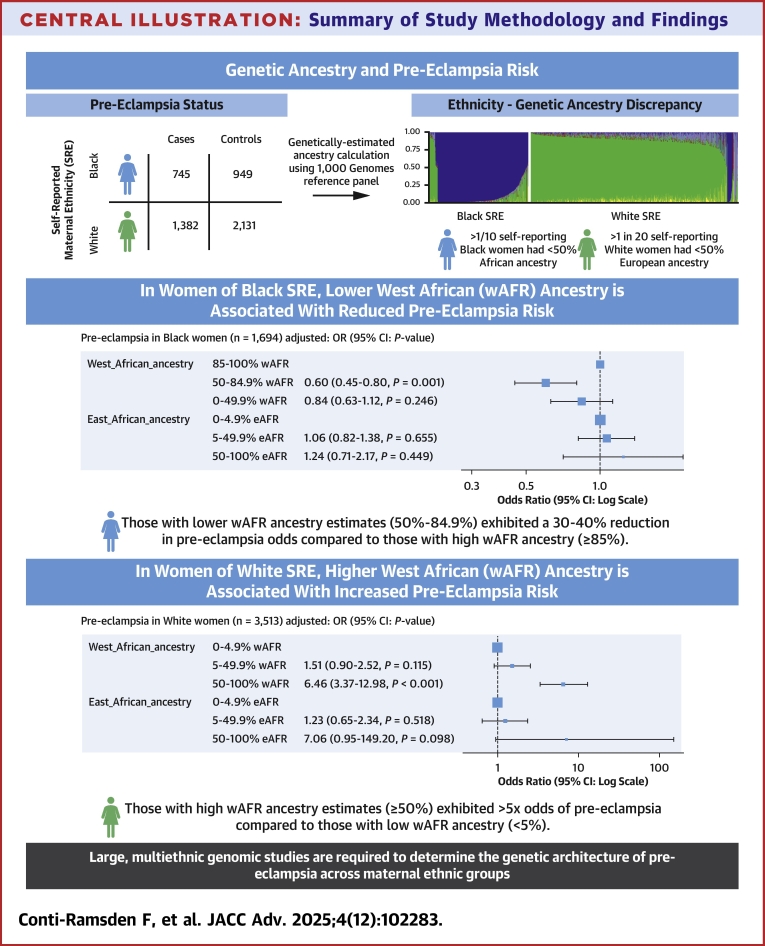
**Summary of Study Methodology and Findings** (Top) Overview of case-control study design and discrepancy between self-reported ethnicity vs genetically computed ancestry estimates. (Bottom) Summary of adjusted association between West African genetic ancestry and pre-eclampsia risk in women of self-reported Black and White ethnic backgrounds.

### Comparison of maternal ethnicity and genetic individual ancestry estimates

**Figure 1:**
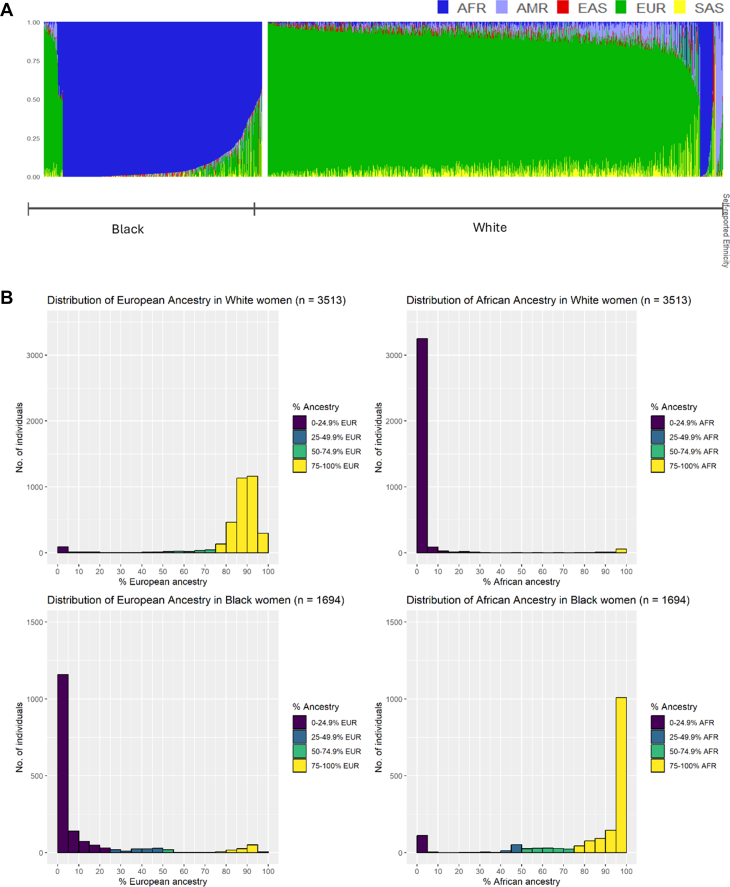

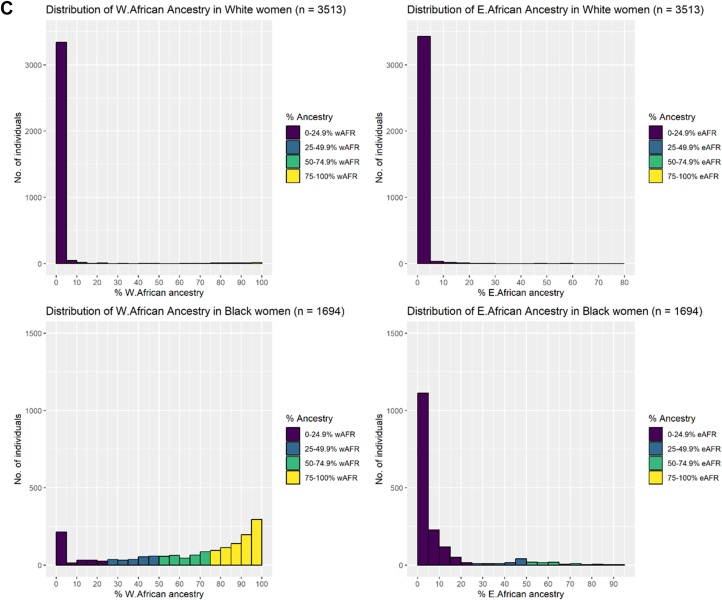
**Genetically Computed Ancestry Estimates** A) Genetic individual ancestry estimate proportions (AFR: African, AMR: Admixed American (Hispanic), EAS: East Asian, EUR: European, SAS: South Asian superpopulations). Each individual is denoted by a single vertical stacked bar, with individual population ancestry percentages (summing to 1) denoted by bar coloring. B) Histograms of African and European genetic individual ancestry estimate percentages. C) Histograms of West (W) and East (E) African genetic individual ancestry estimate percentages. Study group individuals are grouped by self-reported ethnic group throughout. wAFR = West African.

Genetic individual superpopulation ancestry estimates (EUR, AFR, SAS, EAS, and AMR superpopulations) stratified by self-reported maternal ethnicity are shown in Figure 1A. EUR and AFR superpopulation ancestries were the predominant genetic ancestries in the cohort, with distribution of these ancestries across individuals of self-reported White and Black ethnic backgrounds illustrated in Figure 1B, and distribution of regional AFR ancestries illustrated in Figure 1C. Population-level genetic individual ancestry estimates (eg, Yoruba, British) are illustrated in Supplemental Figure 2, with detailed population codes and information available in Supplemental Table 1. The most common AFR ancestries among individuals of self-reported Black ethnic backgrounds (median percentage 1% or more across individuals) were the following 1000 Genomes reference groups: Yoruban in Nigeria; Esan in Nigeria, Mende in Sierra Leone, and Luhya in Kenya (Supplemental Figure 2). With the exception of Luyha (East AFR), these are all West AFR ancestries.

**Figure 2:**
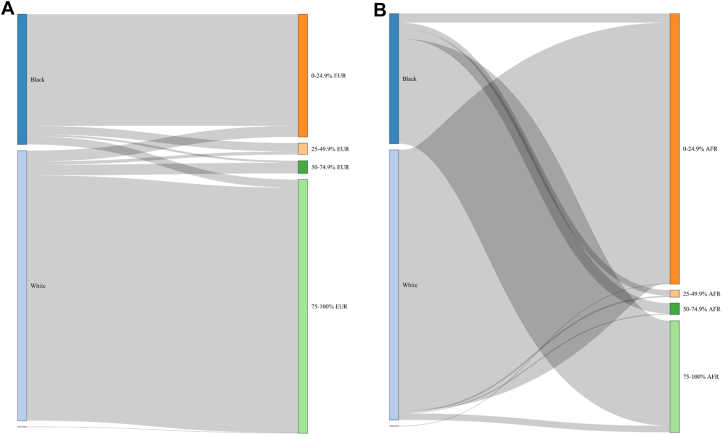
**Comparison of Self-Reported Ethnicity and Genetically Computed Ancestry Estimates** Sankey plot illustrating relationship between self-reported maternal ethnic group (left side) and genetically computed individual ancestry estimates (right side) for A) European (EUR) and B) African (AFR) genetic ancestry.

Although self-reported ethnic group generally aligned with corresponding high percentage EUR or AFR superpopulation genetic individual ancestry estimates (Figure 1), there was varying discrepancy between SRE and genetic ancestry in both groups (Figure 2). Discrepancy between SRE and genetic individual ancestry estimates (at thresholds of <25%, <50%, and <75% expected genetic ancestry) are summarized in Table 2. Across all thresholds, ethnicity-ancestry discrepancy was higher in women of self-reported Black as opposed to White backgrounds. At a threshold of <50%, 11.3% of self-reported Black women (95% CI: 9.9% to 12.9%) and 5.2% of self-reported White women (95% CI: 4.5% to 6.0%) had ethnicity-ancestry discrepancy (Table 2). At thresholds of <25% and <75%, respectively, ethnicity-ancestry discrepancy was 7.0% and 19.3% in self-reported Black women and 4.1% and 9.3% for self-reported White women (Table 2). A small proportion of women of self-reported Black and White ethnic backgrounds also had a high percentage (50% or more) AMR genetic individual ancestry estimates (1.5% and 0.5%, respectively) (Table 2). EAS and SAS genetic ancestries were rare in this study cohort, with 10 or fewer individuals with genetic individual ancestry estimates percentages of 50% or more for either of these ancestries.

**Table 2:** Cross-Tabulation of Self-Reported Ethnicity

With Percentage EUR Genetic Individual Ancestry Estimate Group			
Self-Reported Ethnicity	% European Ancestry	Count (n)	Percentage (%)
White, N = 3,513	0%-24.9% EUR	143	4.1
	25%-49.9% EUR	41	1.2
	50%-74.9% EUR	139	4.0
	75%-100% EUR	3,190	90.8
Black, N = 1,694	0%-24.9% EUR	1,453	85.8
	25%-49.9% EUR	109	6.4
	50%-74.9% EUR	27	1.6
	75%-100% EUR	105	6.2

With Percentage AFR Genetic Individual Ancestry Estimate Group			
Self-Reported Ethnicity	% African Ancestry	Count (n)	Percentage (%)
White, N = 3,513	0%-24.9% AFR	3,398	96.7
	25%-49.9% AFR	18	0.5
	50%-74.9% AFR	13	0.4
	75%-100% AFR	84	2.4
Black, N = 1,694	0%-24.9% AFR	119	7.0
	25%-49.9% AFR	73	4.3
	50%-74.9% AFR	135	8.0
	75%-100% AFR	1,367	80.7

With Percentage AMR Genetic Individual Ancestry Estimate Group			
Self-Reported Ethnicity	% Admixed American Ancestry	Count (n)	Percentage (%)
White, N = 3,513	0%-24.9% AMR	3,432	97.7
	25%-49.9% AMR	29	0.8
	50%-74.9% AMR	14	0.4
	75%-100% AMR	38	1.1
Black, N = 1,694	0%-24.9% AMR	1,685	99.5
	25%-49.9% AMR	7	0.4
	50%-74.9% AMR	0	0.0
	75%-100% AMR	2	0.1

AFR = African; AMR = Admixed American; EUR = European.

### Association between African genetic individual ancestry estimates and pre-eclampsia

Among self-reported White women, the median West AFR genetic ancestry was 0.004% (Q1-Q3: 0.004%-1.370%), and median East AFR genetic ancestry was 0.001% (Q1-Q3: 0.001%-0.001%), reflecting very low AFR ancestry in most women in this group (Figure 1C). As such, thresholds of <5% West and <5% East AFR ancestries were selected to define the reference group, excluding individuals with any meaningful percentage AFR admixture. Given the broader distribution of West AFR genetic ancestry among self-reported Black women (median 74.3%, Q1-Q3: 39.4%-92.0%) (Figure 1C), a cutoff of ≥85% was selected to define the reference group, capturing individuals with a high West AFR genetic ancestry. In contrast, there were very few women with high proportions of East AFR ancestry among self-reported Black women (median 1.8%, Q1-Q3: 0.001%-7.9%); therefore, <5% East AFR ancestry was defined as the reference group. Furthermore, a 50% threshold was applied to genetic ancestry distributions to generate 3 groups, balancing the need for granular ancestry classification with sufficient cell sizes for statistical inference.

**Figure 3:**
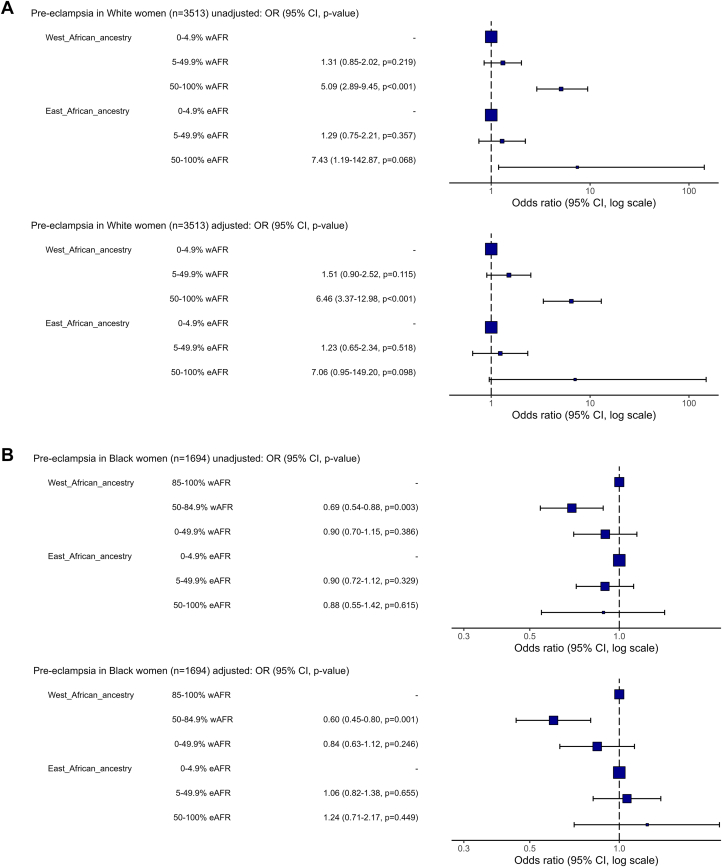
**Association Between Genetically Computed Ancestry and Pre-Eclampsia Risk** Forest plots demonstrating ORs for association of West and East African genetic ancestry percentage categories and pre-eclampsia risk in unadjusted and adjusted models (models adjusted for the following covariates: mean arterial pressure, maternal age, body mass index, smoking status, conception, diabetes, previous pre-eclampsia, and family history of pre-eclampsia), stratified by self-reported maternal ethnicity.

In women of self-reported White ethnic backgrounds, high West AFR individual genetic ancestry percentage category (50% to 100%) was associated with an increased risk of PE compared to those with low West AFR (reference: <5%, OR: 5.09; 95% CI: 2.89-9.45; *P* < 0.001), with consistent effects in models adjusted for MAP, maternal age, BMI, smoking status, conception method, diabetes, previous PE, and family history of PE (aOR: 6.46; 95% CI: 3.37-12.98; *P* < 0.001) (Figure 3, Supplementary Table 2). Small numbers of White women with East AFR genetic ancestries precluded interpretation of these associations. In women of self-reported Black ethnic backgrounds, women with lower West AFR genetic ancestry percentage (50%-84.9%) had a lower PE risk than women with high West AFR genetic ancestry percentage category (reference: 85%-100%) across unadjusted (OR: 0.69; 95% CI: 0.54-0.88; *P* = 0.003) and adjusted models (aOR: 0.60; 95% CI: 0.45-0.80; *P* < 0.001) (Figure 3, Supplemental Table 2). There was no association between East AFR genetic ancestry category and PE in self-reported Black women, and this analysis was similarly limited by small numbers of women with East AFR genetic ancestry.

Sensitivity analysis excluding women with high ethnicity-ancestry discordance (self-reported White ethnicity with <10% EUR ancestry (n = 105); self-reported Black ethnicity with <10% AFR ancestry (n = 116), demonstrated results were sensitive to inclusion of highly discrepant individuals; in self-reported White women exclusion of women with very low EUR ancestry led to a similar pattern of results but associations were no longer statistically significant (Supplemental Figure 3). In self-reported Black women results remained statistically significant and women in the lowest West AFR group (0% to 49.9%) additionally had a lower risk of PE compared to those with high West AFR ancestry (85% to 100%) (Supplemental Figure 3).

### Addition of west African genetic individual ancestry estimates to pre-eclampsia prediction models

**Figure 4:**
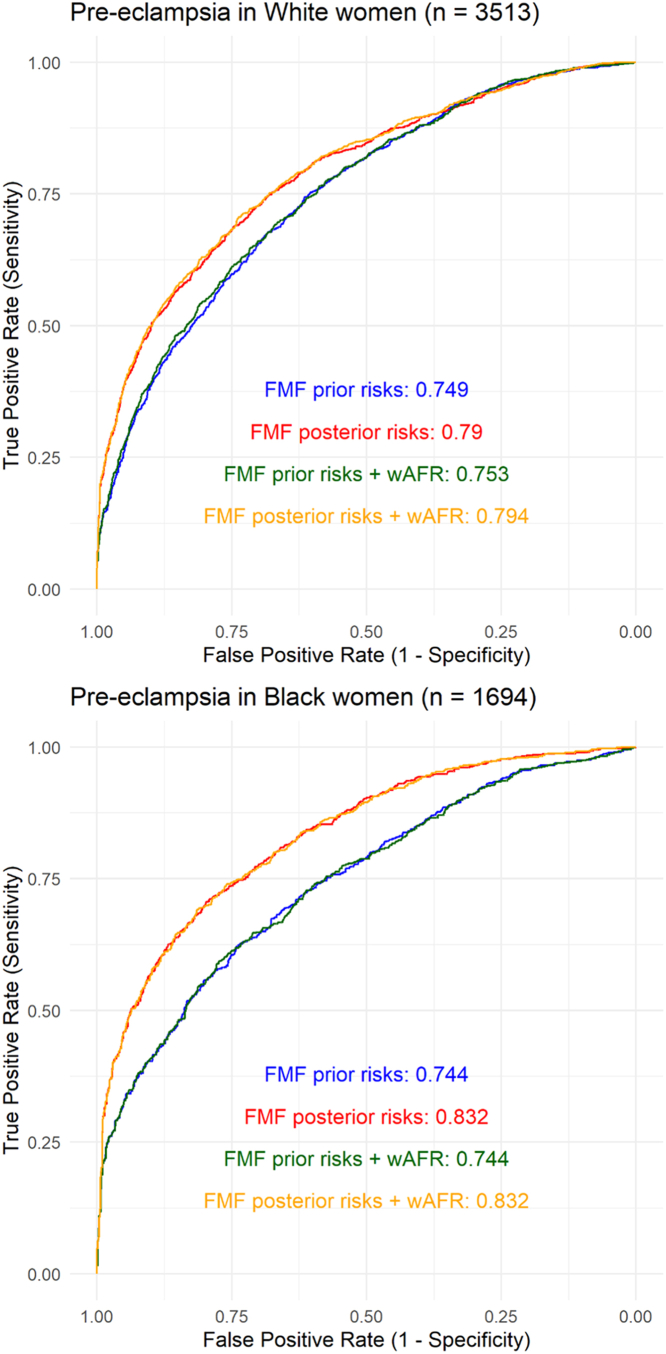
**Pre-Eclampsia Prediction Model Performance With and Without Genetically Computed Ancestry** Area under the receiver operator curve (AUROC) for outcome of all pre-eclampsia predicted by FMF prior (clinical risk factors only) and FMF algorithm posteriors (clinical risk factors, mean arterial pressure, uterine artery pulsatility index, and PAPP-A) with and without addition of percentage West African genetic individual ancestry estimates (wAFR) in women of self-reported White and Black ethnicity. FMF = Fetal Medicine Foundation; other abbreviations as in Figure 1.

Baseline performance of the FMF algorithm (prior and posterior risks) was assessed in women of self-reported Black and White ethnicity, demonstrating high AUROC for both groups (Figure 4). Addition of West AFR genetic individual ancestry estimates to prior (AUROC: 0.749) and posterior (AUROC: 0.790) FMF predictions in women of White ethnic backgrounds did not increase AUROC for PE prediction (Figure 4) (prior model increase: 0.0037; 95% CI: −0.0005-0.0080; *P* = 0.087, posterior model increase: 0.0035; 95% CI: −0.0009-0.0079; *P* = 0.12). In women of Black ethnic backgrounds, addition of genetically estimated West AFR ancestry category to prior (AUROC: 0.744) and posterior (AUROC: 0.832) also did not increase AUROC (Figure 4) (prior model increase: 0.0005; 95% CI: −0.0026-0.0017; *P* = 0.67, posterior model increase: 0.0000; 95% CI: −0.0022-0.0022; *P* > 0.99).

**Figure 5:**
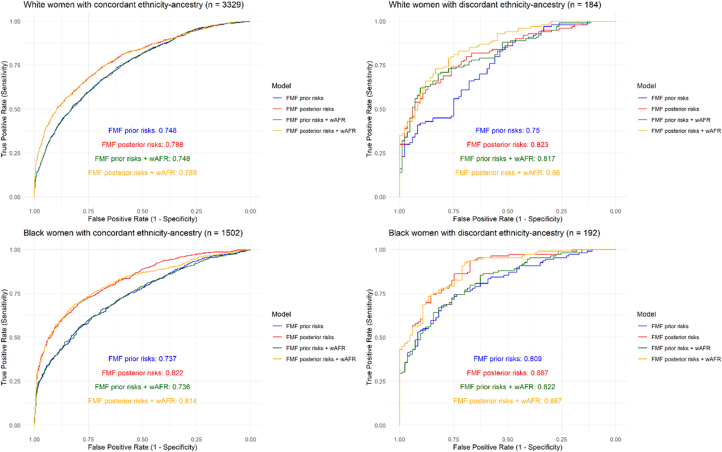
**Pre-Eclampsia Prediction Model Performance in Ethnicity-Ancestry Discordant Individuals** Area under the receiver operator curve (AUROC) for outcome of all pre-eclampsia predicted by FMF prior (clinical risk factors only) and FMF algorithm posteriors (clinical risk factors, mean arterial pressure, uterine artery pulsatility index, and PAPP-A) with and without addition of percentage West African genetic individual ancestry estimates (wAFR) in women of self-reported White and Black ethnicity stratified by ethnicity-ancestry discrepancy. Abbreviations as in Figures 1 and 4.

To further explore the utility of genetically estimated ancestry in early pregnancy prediction of PE, ethnic groups were stratified according to discrepancy between ethnicity and genetic individual ancestry estimates (with discrepancy defined as SRE White and <50% EUR genetically estimated ancestry or SRE Black and <50% AFR genetically estimated ancestry). In both Black and White women with concordant ethnicity-ancestry (n = 1502 Black and n = 3329 White), addition of genetically estimated West AFR ancestry did not affect PE prediction model performance (Figure 5).

In women of self-reported White ethnicity with discrepant ethnicity-ancestry (n = 184), the addition of West AFR genetic individual ancestry estimates significantly improved the performance of the FMF prior (AUROC: 0.750) but not the posterior risk model (AUROC: 0.823) (prior model increase: 0.0668; 95% CI: 0.0070-0.1266; *P* = 0.029. posterior model increase: 0.0370; 95% CI: −0.0135-0.0875; *P* = 0.15) (Figure 5). In contrast, among women of self-reported Black ethnicity with discordant ancestry (n = 192), inclusion of West AFR genetic ancestry estimates did not significantly improve either prior (AUROC: 0.809) or posterior (AUROC: 0.887) risk model performance (prior model increase: +0.0130; 95% CI: −0.0137-0.0398; *P* = 0.34, posterior model increase: 0.0002; 95% CI: −0.0151-0.0155; *P* = 0.98).

## Discussion

This is the largest multiethnic case-control study to date to directly compare self-reported maternal ethnicity with genetic estimates of individual ancestry in the context of PE risk and prediction. We show that SRE often diverges from underlying genetic individual ancestry estimates, with over 10% of self-reporting Black women and over 5% of self-reporting White women having <50% of the continental ancestry typically associated with their reported ethnicity. Importantly, we demonstrate that higher West AFR genetic ancestry is independently associated with increased risk of PE, even after adjusting for established clinical risk factors including maternal age, BMI, prior history of PE, and baseline BP.

Among women who self-identified as White, those with high West AFR genetic individual ancestry estimates (≥50%) exhibited a >5-fold increased odds of PE compared to those with low West AFR ancestry (<5%). Similarly, among women who self-identified as Black, those with lower West AFR genetic individual ancestry estimates (50%-84.9%) demonstrated a 30% to 40% reduction in PE odds compared to those with high West AFR ancestry (≥85%). These findings highlight the limitations of using SRE as a surrogate for biological risk and support the potential role of genetic ancestry in refining PE risk stratification.

However, when genetic individual ancestry estimates were added to the FMF PE risk algorithm, it did not significantly improve model performance. This may reflect the fact that genetic ancestry partially underlies variation in key predictors already incorporated into the model—including maternal ethnicity but also family history of PE, history of chronic hypertension, BP, uterine artery Doppler indices, and biochemical markers—thereby limiting the additional predictive value of ancestry data beyond these established clinical and biophysical variables. In addition, although ethnicity-ancestry discrepancy was observed in our study, the majority of individuals (90%-95%) demonstrated congruence between SRE and genetic ancestry, potentially limiting the impact on prediction model performance. The utility of adding genetic ancestry estimates to the FMF algorithm may vary across populations with different admixture levels. We hypothesize that such additions would provide the greatest benefit in highly admixed populations and may become more beneficial in the future as global populations become increasingly admixed.

### Study Strengths and limitations

This study leverages a large, well-characterized, multiethnic cohort to examine the relationship between genetically estimated ancestry and PE risk and includes the largest genotyped population of women with PE who self-identify as Black (n = 745). It builds on our prior work in a smaller cohort (n = 436),^17^ offering greater statistical power and allowing for granular analyses of ancestry-ethnicity discrepancy and of reference ancestry subgroups. Notably, the largest Genome-Wide Association Study and meta-analysis for PE to date included just 20 cases in women of Black ethnic backgrounds of a total of 17,150 cases.^27^ Although we did not have a sufficient sample size for Genome-Wide Association Study, the use of a genetic ancestry approach enabled us to find statistically significant association between West AFR genetically estimated ancestry and risk of PE, and this study further represents an important step forward in generating genetic data to address Eurocentric bias in contemporary genomics.^28^

We did not perform an a priori sample size calculation; all women with a clinical diagnosis of PE who had given consent to genetic studies in the HBC were genotyped. West AFR (Yoruba, Esan, and Mende) genetically estimated ancestries were the most prevalent AFR ancestries in the study group, consistent with the predominant ethnic origin of British individuals of AFR ancestry. Although the largest multiethnic study of PE to date, numbers are modest in comparison to the cohorts used to develop the FMF algorithm (n ∼60,000)^11^ and as such, for the lower prevalence East AFR population ancestry in this study, we were underpowered to detect associations. Further investigation of multiethnic cohorts in future genetic studies will enable more definitive conclusions to be drawn on the association of AFR, genetic variants, and PE disease risk.

Although the optimal definition and computation of genetically estimated individual ancestry remains a subject of debate,^29^^,^^30^ individual genetic ancestry estimation using reference multiancestral populations as reference groups enabled precise, reproducible measurements of the components of ancestry.^17^ An additional strength of this study is the granularity and quality of phenotyping, demonstrated by low rates of missing data, availability of established PE clinical risk factors inputted into adjusted models, and diagnosis of PE made by trained clinicians using robust definitions from international guidelines.^31^ This contrasts with the majority of genetic studies of PE which use electronic health records and International Classification of Diseases-10 codes and have limited clinical risk factors available for adjusted models.^27^ We took care to exclude the potential for ethnic group misclassification alone driving results; self-reported ethnic group in the study data set was compared to the ethnic group recorded in the electronic health record where available with discrepancy observed in just 1% of cases. Exclusion of highly discrepant individuals in a sensitivity analysis yielded a similar overall pattern of results. Among Black women this exclusion strengthened the association between lower West AFR ancestry and reduced PE risk. In White women, the direction of association remained consistent, but it was no longer statistically significant, reflecting the relatively small number of women with West AFR population ancestry in this group.

### Interpretation in light of other studies

In our previous cohort study of 436 women across 2 sites, we observed a substantially higher rate of discrepancy between SRE and genetically estimated individual ancestry estimates at a threshold of 50%—approximately 10% among women identifying as White and 20% among those identifying as Black.^17^ We speculate that the more rigorous, prospective, clinician-led recruitment and phenotype ascertainment in the curated Harris Birthright Centre data set may partially account for these differences. However, the higher discrepancy observed in our previous study may more accurately reflect real-world patterns in contemporary clinical, multiethnic obstetric populations given the complexity in defining ethnicity.^32^ Discrepancy between SRE and genetically estimated individual ancestry is expected to become more common as global populations become increasingly admixed, and rigid ethnic categories become less adequate for describing an individual’s complex, continuous genetic ancestry. In keeping with this, the National Academies of Sciences, Engineering and Medicine and the American Journal of Human Genetics, both recommend that SRE should not be used as a proxy for genetic ancestral groups and encourage the reporting and use of genetic ancestry in genomic studies.^33^

To our knowledge, our previous study and a study of 125 women with gestational hypertension or PE or hemolysis, elevated liver enzymes, and low platelets syndrome or controls (n = 161) in Hispanic women living in the United States^34^ are the only studies using a genetic ancestry approach in PE. Across all 3 studies, AFR genetically estimated ancestry proportion was associated with a higher risk of hypertensive disorders of pregnancy or PE in adjusted models, with our previous and current study further suggesting this is potentially specific to West AFR ancestries although confirmation in larger studies is warranted. Consistency of effects across geographies, populations, and timing of onset of PE lends credibility to the findings of this study.

### Clinical relevance and future studies

Our findings of ethnicity-ancestry discrepancy support critiques of using self-identified ethnicity as a reliable proxy for biological risk. This suggests reconsideration of ethnicity groupings in PE risk prediction. UK NICE (National Institute for Health and Care Excellence)^35^ and U.S. ACOG^36^ guidelines recommend clinical risk factor screening for PE, with additional international guidelines endorsing multivariable prediction tools where resources permit (ISSHP [International Society for the Study of Hypertension in Pregnancy],^31^ SOMANZ [Society of Obstetric Medicine of Australia and New Zealand],^37^ and SOGC^38^). Currently only ACOG guidelines identify Black ethnicity as an independent clinical risk factor for PE, noting that the U.S. Preventative Services Task Force includes Black ethnicity as a moderate risk factor representing societal racism rather than biological predisposition.^39^ This work highlights that alongside investigation of racism and social determinants of health as contributors to PE risk in women of Black ethnic backgrounds, investigation of biological pathways is also necessary and may have important implications for clinical risk assessment. Previous work has also shown that self-reported maternal ethnicity affects PE biomarker (placental growth factor/soluble Flt-1) distributions, with implications for diagnostic performance across maternal ethnic groups.^40^ The broad “Black” ethnic category currently incorporated into the FMF algorithm, including for biomarker adjustment, and other clinical risk prediction tools may merit reconsideration. As molecular insights into PE evolve across diverse populations, incorporating more specific variables, such as self-reported ancestry or genetically defined markers, may enhance the biological relevance and clinical accuracy of risk assessment.

Our study findings also provide strong rationale for large, multiethnic genomic studies of PE, prioritizing inclusion of women of Black ethnic backgrounds and AFR ancestry. This is likely to drive further insights into the genetic architecture of this complex phenotype. Our study also adds further weight to the possibility that variants specific to West AFR ancestry populations such as apolipoprotein 1, increasingly implicated in kidney and cardiovascular disease,^41^^,^^42^ may be associated with PE disease risk, a hypothesis that is currently being explored.^43^ Although currently addition of genetic ancestry information to the FMF prediction model has limited impacts on model performance, this is likely to evolve as we gain increasing understanding of the molecular pathways driving PE risk across populations and move from broad genetic summary measures (such as genetic ancestry) to precision, multiethnic polygenic scores, and variants.

## Conclusions

Our study confirms maternal ethnicity is an imperfect proxy for genetically estimated individual ancestry, particularly for women of minority ethnic backgrounds. We further demonstrate a strong association between genetically estimated West AFR ancestry and PE risk independent of established risk factors including age, BMI, and baseline BP in self-reported Black and White women. This provides strong rationale for genomic studies in larger, multiethnic, well-phenotyped pregnancy hypertension cohorts to allow further exploration of the association of genetic ancestry estimates with PE risk and enable finer mapping of population-specific causal genomic regions and variants. Disentangling the causal pathways driving the well-established epidemiology of maternal ethnicity and PE risk and understanding potential genomic contributions has the potential to transform our molecular understanding of PE and greatly improve risk prediction, preventative management, and thereby clinical outcomes.

Perspectives **COMPETENCY IN MEDICAL KNOWLEDGE:** More than 1 in 10 women who self-identified as of Black ethnic backgrounds and 1 in 20 who self-identified as of White ethnic backgrounds had <50% of expected genetic ancestry, confirming self-reported maternal ethnicity is an imperfect proxy for genetic estimates of individual ancestry in contemporary UK obstetric populations. West AFR genetically estimated ancestry is associated with PE risk independently of established clinical risk factors including age, BMI, history of previous PE, and baseline BP in women of Black and White self-reported ethnic groups.

**TRANSLATIONAL OUTLOOK:** Inclusion of maternal ethnicity in PE prediction algorithms and screening approaches warrants careful consideration, as SRE does not consistently align with genetically estimated ancestry and may not accurately reflect biological risk. Moreover, discrepancy between ancestry and SRE, already present in a substantial proportion of women, is likely to increase in future generations due to growing global genetic admixture. The findings of this study strongly support investigation of biological mechanisms of PE across maternal ethnic groups. Large genomic studies in AFR, AFR heritage, and other under-represented populations are required to better characterize the multiethnic genetic architecture of PE, enhance risk prediction, and support equitable care.

## Funding support and author disclosures

This study was supported by a grant from the Fetal Medicine Foundation. Conti-Ramsden is supported by the 10.13039/501100000265Medical Research Council (MR/V006835/1, external peer review). Dr Chappell is supported by an NIHR Senior Investigator Award. Dr Hysi is supported by 10.13039/100006312BrightFocus, US (G2021011S) and US NIH (R21AI177219). Dr de Marvao is supported by the 10.13039/501100003123Fetal Medicine Foundation (495237). Conti-Ramsden receives part-time salary contribution as Chief Medical Officer at MEGI Health UK Ltd; holds equity in Nexus Connected Limited where they serve as an advisor; and receives consulting fees for advisory services provided through Option 5 Health Limited, Revena Limited and Gerson Lehrman Group Limited (GLG). All other authors have reported that they have no relationships relevant to the contents of this paper to disclose.

## References

[1] Chappell L.C., Cluver C.A., Kingdom J., Tong S. (2021). Pre-eclampsia. Lancet.

[2] Rolnik D.L., Wright D., Poon L.C. (2017). Aspirin versus placebo in pregnancies at high risk for preterm preeclampsia. N Engl J Med.

[3] Khalil A., Rezende J., Akolekar R., Syngelaki A., Nicolaides K.H. (2013). Maternal racial origin and adverse pregnancy outcome: a cohort study. Ultrasound Obstet Gynecol.

[4] Miranda M.L., Swamy G.K., Edwards S., Maxson P., Gelfand A., James S. (2010). Disparities in maternal hypertension and pregnancy outcomes: evidence from North Carolina, 1994-2003. Public Health Rep.

[5] Tanaka M., Jaamaa G., Kaiser M. (2007). Racial disparity in hypertensive disorders of pregnancy in New York state: a 10-year longitudinal population-based study. Am J Public Health.

[6] Urquia M.L., Glazier R.H., Gagnon A.J. (2014). Disparities in pre-eclampsia and eclampsia among immigrant women giving birth in six industrialised countries. BJOG.

[7] Miller E.C., Zambrano Espinoza M.D., Huang Y. (2020). Maternal race/ethnicity, hypertension, and risk for stroke during delivery admission. J Am Heart Assoc.

[8] O’Gorman N., Wright D., Syngelaki A. (2016). Competing risks model in screening for preeclampsia by maternal factors and biomarkers at 11-13 weeks gestation. Am J Obstet Gynecol.

[9] Wright D., Tan M.Y., O’Gorman N. (2019). Predictive performance of the competing risk model in screening for preeclampsia. Am J Obstet Gynecol.

[10] Wright D., Wright A., Nicolaides K.H. (2020). The competing risk approach for prediction of preeclampsia. Am J Obstet Gynecol.

[11] Wright D., Akolekar R., Syngelaki A., Poon L.C.Y., Nicolaides K.H. (2012). A competing risks model in early screening for preeclampsia. Fetal Diagn Ther.

[12] Townsend R., Khalil A., Premakumar Y. (2019). Prediction of pre-eclampsia: review of reviews. Ultrasound Obstet Gynecol.

[13] Tan M.Y., Wright D., Syngelaki A. (2018). Comparison of diagnostic accuracy of early screening for pre-eclampsia by NICE guidelines and a method combining maternal factors and biomarkers: results of SPREE. Ultrasound Obstet Gynecol.

[14] Sacks D.A., Incerpi M.H. (2024). Of aspirin, preeclampsia, and racism. N Engl J Med.

[15] Borrell L.N., Elhawary J.R., Fuentes-Afflick E. (2021). Race and genetic ancestry in medicine — a time for reckoning with racism. N Engl J Med.

[16] Banda Y., Kvale M.N., Hoffmann T.J. (2015). Characterizing race/ethnicity and genetic ancestry for 100,000 subjects in the genetic epidemiology research on adult health and aging (GERA) cohort. Genetics.

[17] Conti-Ramsden F., de Marvao A., Gill C. (2024). Association of genetic ancestry with pre-eclampsia in multi-ethnic cohorts of pregnant women. Pregnancy Hypertens.

[18] Hypertension in pregnancy (2013). Report of the American college of obstetricians and gynecologists’ task force on hypertension in pregnancy. Obstet Gynecol.

[19] List of ethnic groups - GOV.UK. https://www.ethnicity-facts-figures.service.gov.uk/style-guide/ethnic-groups/.

[20] Illumina Infinium global screening Array-24 kit. https://www.illumina.com/products/by-type/microarray-kits/infinium-global-screening.html.

[21] Marees A.T., de Kluiver H., Stringer S. (2018). A tutorial on conducting genome-wide association studies: quality control and statistical analysis. Int J Methods Psychiatr Res.

[22] Chang C.C., Chow C.C., Tellier L.C.A.M., Vattikuti S., Purcell S.M., Lee J.J. (2015). Second-generation PLINK: rising to the challenge of larger and richer datasets. Gigascience.

[23] Pritchard J.K., Stephens M., Donnelly P. (2000). Inference of population structure using multilocus genotype data. Genetics.

[24] Alexander D.H., Novembre J., Lange K. (2009). Fast model-based estimation of ancestry in unrelated individuals. Genome Res.

[25] IGSR The international genome sample resource. https://www.internationalgenome.org/data.

[26] R Core Team (2023).

[27] Honigberg M.C., Truong B., Khan R.R. (2023). Polygenic prediction of preeclampsia and gestational hypertension. Nat Med.

[28] Fatumo S., Chikowore T., Choudhury A., Ayub M., Martin A.R., Kuchenbaecker K. (2022). A roadmap to increase diversity in genomic studies. Nat Med.

[29] Lewis A.C.F., Molina S.J., Appelbaum P.S. (2022). Getting genetic ancestry right for science and society. Science (1979).

[30] Cerdeña J.P., Grubbs V., Non A.L. (2022). Genomic supremacy: the harm of conflating genetic ancestry and race. Hum Genomics.

[31] Magee L.A., Brown M.A., Hall D.R. (2022). The 2021 international society for the study of hypertension in pregnancy classification, diagnosis & management recommendations for international practice. Pregnancy Hypertens.

[32] Conti-Ramsden F., de Marvao A., Chappell L. (2025). Ethnic disparities in hypertensive disorders of pregnancy. Reproduction.

[33] Feero W.G., Steiner R.D., Slavotinek A. (2024). Guidance on use of race, ethnicity, and geographic origin as proxies for genetic ancestry groups in biomedical publications. Am J Hum Genet.

[34] Shahabi A., Wilson M.L., Lewinger J.P., Goodwin T.M., Stern M.C., Ingles S.A. (2013). Genetic admixture and risk of hypertensive disorders of pregnancy among Latinas in Los Angeles County. Epidemiology.

[35] National Institute for Health and Care Excellence (NICE) (2019).

[36] (2019). ACOG Practice Bulletin No. 203: Chronic Hypertension in Pregnancy. Obstet Gynecol.

[37] Society of Obstetric Medicine Australia and New Zealand (SOMANZ) (2023).

[38] Magee L.A., Smith G.N., Bloch C. (2022). Guideline No. 426: hypertensive disorders of pregnancy: diagnosis, prediction, prevention, and management. J Obstet Gynaecol Canada.

[39] Davidson K.W., Barry M.J., Mangione C.M. (2021). Aspirin use to prevent preeclampsia and related morbidity and mortality. JAMA.

[40] Wright A., von Dadelszen P., Magee L.A. (2023). Effect of race on the measurement of angiogenic factors for prediction and diagnosis of pre-eclampsia. BJOG.

[41] Hung A.M., Shah S.C., Bick A.G. (2022). APOL1 risk variants, acute kidney injury, and death in participants with African ancestry hospitalized with COVID-19 from the million veteran program. JAMA Intern Med.

[42] Gbadegesin R.A., Ulasi I., Ajayi S. (2024). APOL1 Bi- and monoallelic variants and chronic kidney disease in West Africans. N Engl J Med.

[43] Osafo C., Thomford N.E., Coleman J. (2022). APOL1 genotype associated risk for preeclampsia in African populations: rationale and protocol design for studies in women of African ancestry in resource limited settings. PLoS One.

