# Supplementary material for: Genetically Estimated Ancestry and the Risk of Pre-Eclampsia: A Multiethnic Case-Control Study
Source: JACC Adv. 2025 Nov 3;4(12):102283. doi: 10.1016/j.jacadv.2025.102283 (PMC12793860; doi:10.1016/j.jacadv.2025.102283)

Supplementary materials

Supplementary Table 1. Population and superpopulation codes and descriptions of 1000 Genome individuals. The populations in the 1000 Genomes Project were selected to represent global human genetic diversity. The study aimed to capture a wide variety of genetic variations across different ethnic and geographic groups. The participants were chosen from 26 populations spread across 14 different countries, covering diverse regions including sub-Saharan Africa, Europe, East Asia, South Asia, and the Americas. These populations were selected to reflect historical migration patterns and to ensure broad representation of human genetic variation.

**Population Code**	**Population Description**	**Super Population Code**
CHB	Han Chinese in Beijing, China	EAS: East Asian
JPT	Japanese in Tokyo, Japan	EAS
CHS	Southern Han Chinese	EAS
CDX	Chinese Dai in Xishuangbanna, China	EAS
KHV	Kinh in Ho Chi Minh City, Vietnam	EAS
CEU	Utah Residents (CEPH) with Northern and Western European Ancestry	EUR: European
TSI	Toscani in Italia	EUR
FIN	Finnish in Finland	EUR
GBR	British in England and Scotland	EUR
IBS	Iberian Population in Spain	EUR
YRI	Yoruba in Ibadan, Nigeria	AFR: African
LWK	Luhya in Webuye, Kenya	AFR
GWD	Gambian in Western Divisions in the Gambia	AFR
MSL	Mende in Sierra Leone	AFR
ESN	Esan in Nigeria	AFR
ASW	Americans of African Ancestry in SW USA	AFR
ACB	African Caribbeans in Barbados	AFR
MXL	Mexican Ancestry from Los Angeles USA	AMR: Admixed American
PUR	Puerto Ricans from Puerto Rico	AMR
CLM	Colombians from Medellin, Colombia	AMR
PEL	Peruvians from Lima, Peru	AMR
GIH	Gujarati Indian from Houston, Texas	SAS: South Asian
PJL	Punjabi from Lahore, Pakistan	SAS
BEB	Bengali from Bangladesh	SAS
STU	Sri Lankan Tamil from the UK	SAS

Population Code	Population Description	Super Population Code
ITU	Indian Telugu from the UK	SAS

Supplementary Table 2. Unadjusted and adjusted (models adjusted for the following covariates: *mean arterial pressure, maternal age, body mass index, smoking status, conception, diabetes, previous pre-eclampsia and family history of pre-eclampsia*) logistic regression models of association between West and East African genetically computed individual ancestry estimates and outcome of all pre-eclampsia stratified by maternal self-reported ethnicity. *Odds ratios (OR) and adjusted OR (aOR) and 95% confidence intervals (CI) are presented for categorized genetically-computed individual ancestry estimate groups to allow for non-linearity of associations, with the appropriate ancestry group set as reference.*

Genetic ancestry % category	N	OR [95% CI]	p-value	aOR [95% CI]	p-value
**Ethnicity: self-reported White (n = 3513)**					
**West African**					
Reference: 0-4.9%	3341				
5-49.9%	101	1.31 [0.85-2.02]	0.22	1.51 [0.90-2.52]	0.11
50-100%	71	**5.09 [2.89-9.45]**	**<0.001**	**6.46 [3.37-12.98]**	**<0.001**
**East African**					
Reference: 0-4.9%	3429				
5-49.9%	78	1.29 [0.75-2.21]	0.36	1.23 [0.65-2.34]	0.52
50-100%	6	7.43 [1.19-142.87]	0.068	7.06 [0.95-149.20]	0.098
**Ethnicity: self-reported Black (n = 1694)**					
**West African**					
0-49.9%	540	0.90 [0.70-1.15]	0.39	0.84 [0.63-1.12]	0.25
50-84.9%	524	**0.69 [0.54-0.88]**	**0.003**	**0.60 [0.45-0.80]**	**<0.001**
Reference: 85-100%	630				

**East African**					
Reference: 0-4.9%	1112				
5-49.9%	498	0.90 [0.72-1.12]	0.33	1.06 [0.82-1.38]	0.65
50-100%	84	0.88 [0.55-1.42]	0.61	1.24 [0.71-2.17]	0.45

Supplementary Figure 1. Study flow diagram.

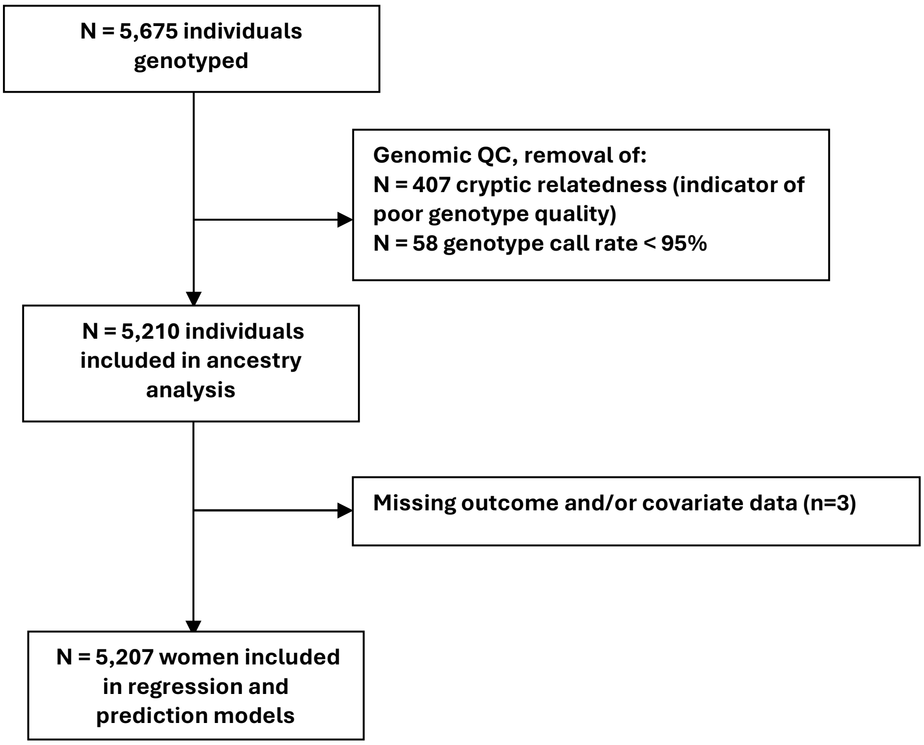

Supplementary Figure 2. Genetic individual ancestry estimate proportions in whole study cohort (n = 5210). Each individual is denoted by a single vertical stacked bar, with individual population ancestry percentages (summing to 1) denoted by bar colouring. Individuals are grouped by self-reported ethnic group. Population-level ancestry estimates are displayed, with ancestries with median percentage across Black or White individuals of 1% or more displayed individually in graph (CEU: Northern/Western European in Utah, ESN: Esan in Nigeria, FIN: Finnish in Finland, GBR: British in England and Scotland, IBS: Iberian Population in Spain, LWK: Luhya in Webuye, Kenya, MSL: Mende in Sierra Leone, TSI: Toscani in Italia, YRI: Yoruba in Ibadan, Nigeria), and all other population-level ancestries aggregated into a single 'other' label.

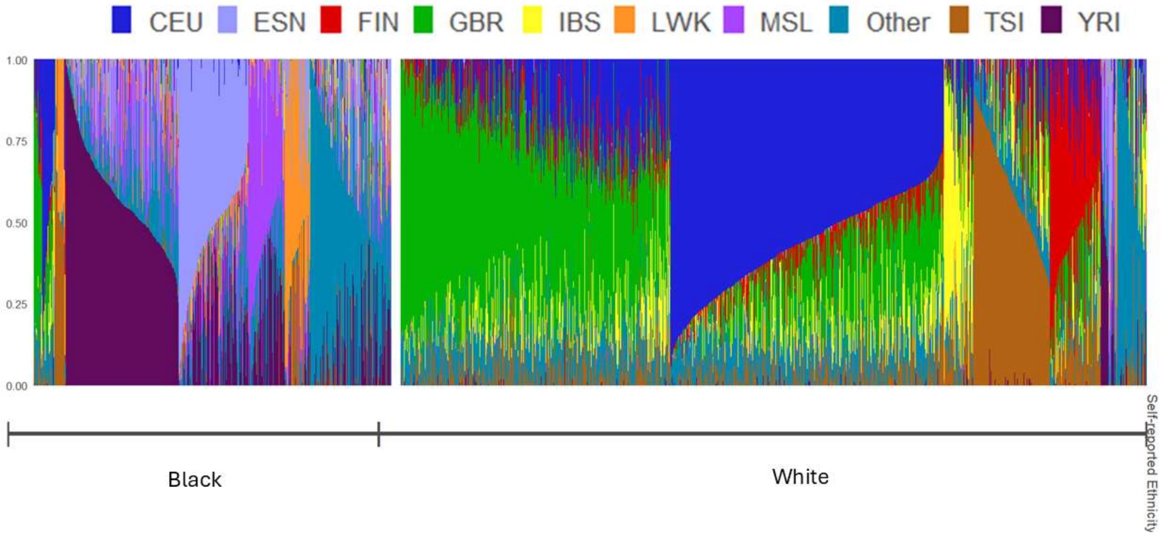

Supplementary Figure 3. Sensitivity analysis forest plots demonstrating odds ratios for association of West and East African genetic ancestry percentage categories and pre-eclampsia risk in unadjusted and adjusted models (models adjusted for the following covariates: *mean arterial pressure, maternal age, body mass index, smoking status, conception, diabetes, previous pre-eclampsia and family history of pre-eclampsia*), stratified by self-reported maternal ethnicity, where highly ethnicity-ancestry discrepant individuals were removed from analysis (defined as: self-reported white and < 10% European ancestry, self-reported Black and < 10% African ancestry).

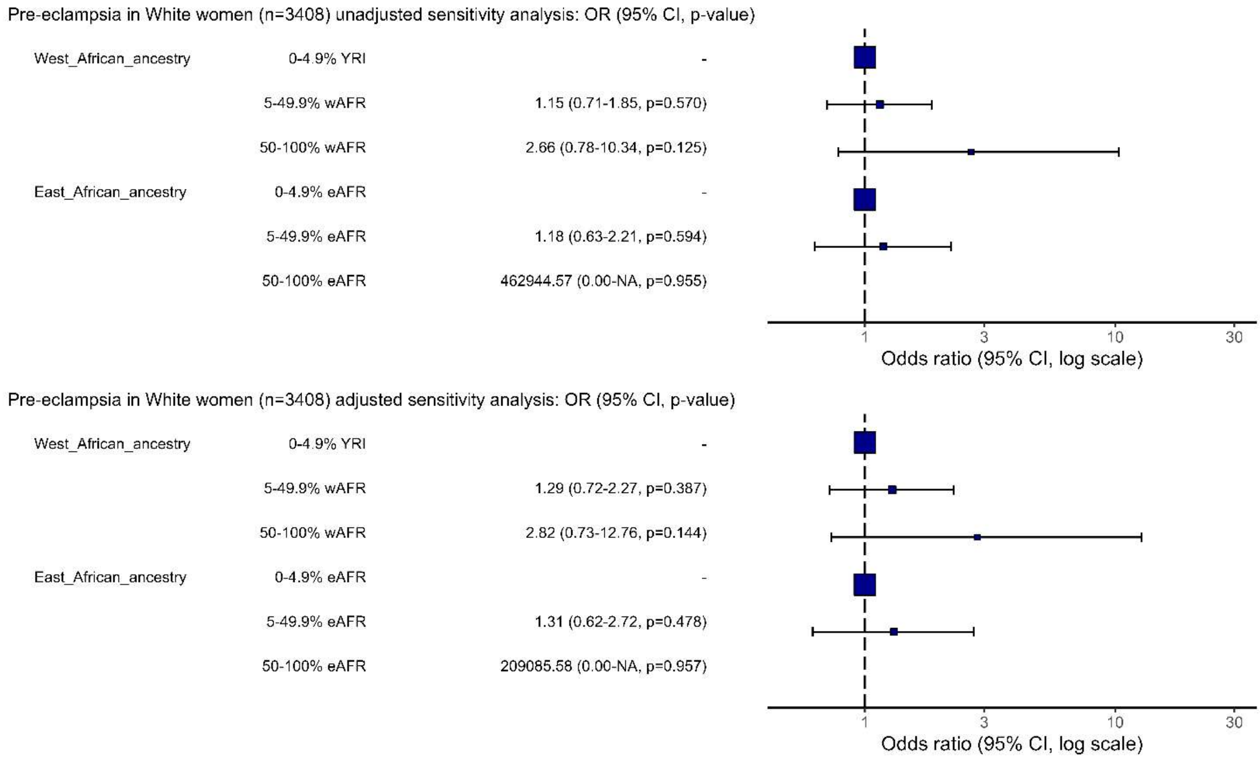

Pre-eclampsia in Black women (n=1578) unadjusted sensitivity analysis: OR (95% CI, p-value)

West_African_ancestry	85-100% wAFR	-
	50-84.9% wAFR	0.66 (0.51-0.84, p=0.001)
	0-49.9% wAFR	0.57 (0.43-0.75, p<0.001)
East_African_ancestry	0-4.9% eAFR	-
	5-49.9% eAFR	1.09 (0.86-1.37, p=0.484)
	50-100% eAFR	1.45 (0.88-2.37, p=0.147)

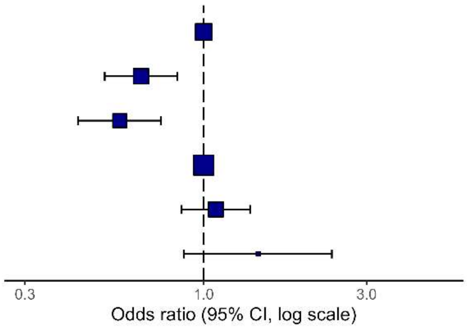

Pre-eclampsia in Black women (n=1578) unadjusted sensitivity analysis: OR (95% CI, p-value)

West_African_ancestry	85-100% wAFR	-
	50-84.9% wAFR	0.57 (0.43-0.76, p<0.001)
	0-49.9% wAFR	0.55 (0.39-0.76, p<0.001)
East_African_ancestry	0-4.9% eAFR	-
	5-49.9% eAFR	1.25 (0.96-1.64, p=0.099)
	50-100% eAFR	1.94 (1.08-3.47, p=0.026)

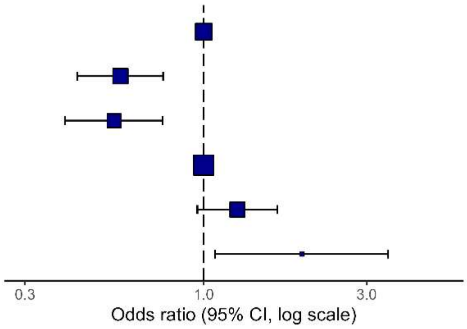

Supplement: Supplemental Tables S1 and S2 and Supplemental Figures S1 to S3. [file mmc1.pdf]
